# The Rational Design of Therapeutic Peptides for Aminopeptidase N using a Substrate-Based Approach

**DOI:** 10.1038/s41598-017-01542-5

**Published:** 2017-05-02

**Authors:** Shilvi Joshi, Lang Chen, Michael B. Winter, Yi-Lun Lin, Yang Yang, Mariya Shapovalova, Paige M. Smith, Chang Liu, Fang Li, Aaron M. LeBeau

**Affiliations:** 1 Department of Pharmacology, University of Minnesota Medical School, Minneapolis, MN 55455 USA; 2 Department of Pharmaceutical Chemistry, University of California, San Francisco, CA 94153 USA

## Abstract

The M1 family of metalloproteases represents a large number of exopeptidases that cleave single amino acid residues from the N-terminus of peptide substrates. One member of this family that has been well studied is aminopeptidase N (APN), a multifunctional protease known to cleave biologically active peptides and aide in coronavirus entry. The proteolytic activity of APN promotes cancer angiogenesis and metastasis making it an important target for cancer therapy. To understand the substrate specificity of APN for the development of targeted inhibitors, we used a global substrate profiling method to determine the P1–P4′ amino acid preferences. The key structural features of the APN pharmacophore required for substrate recognition were elucidated by x-ray crystallography. By combining these substrate profiling and structural data, we were able to design a selective peptide inhibitor of APN that was an effective therapeutic both *in vitro* and *in vivo* against APN-expressing prostate cancer models.

## Introduction

The M1 aminopeptidase family is a group of Zn^2+^-dependent peptidases expressed ubiquitously by both fetal and adults tissues. Protein levels of M1 aminopeptidases have been documented in the brain, pancreas, lung, intestines, prostate, heart, endothelial cells and in components of the immune system^1, 2^. Each M1 aminopeptidase demonstrates unique substrate specificity by preferring certain amino acids at the N-terminus of their endogenous substrates. For example, aminopeptidase B prefers basic amino acids, whereas aminopeptidase A prefers acidic amino acids^3^. The substrate specificities of the aminopeptidases allow each of them to selectively catalyze the activation or metabolism of bioactive peptides. The most studied member of the mammalian M1 aminopeptidase family is aminopeptidase N (APN), also known as CD13. APN exists as a dimeric 110 kDa cell surface protein with a small N-terminal intracellular domain, a single-pass transmembrane anchor, a small extracellular stalk, and a large ectodomain on the C-terminus^4^. Cleaving after neutral amino acids, as implied by the “N” in its name, APN degrades peptides that are involved in different physiological pathways, including pain sensation and mood disorder by inactivating enkephalin, as well as regulating blood pressure by cleaving angiotensin III^1, 5^. APN is considered to be a “moonlighting ectoenzyme”, possessing functions other than its role as a peptidase^1^. Independent of its enzymatic activity, APN can also act a receptor for viral infection and as an adhesion molecule^6^.

In cancer, APN is widely over-expressed on the surface of a number of different cell types, ranging from endothelial cells to solid tumor cells. Enzymatically active APN has been documented to play import roles in tumorigenesis, angiogenesis, cell migration, and metastasis^7–9^. As a result of its role in cancer development and metastasis, APN has been a major target for drug development. The direct enzymatic activity of APN has been targeted using the potent transition-state analogue inhibitor bestatin (ubenimex) in several clinical trials^8, 10^. Although bestatin inhibits nearly a dozen aminopeptidases, it has demonstrated therapeutic benefit in acute myeloid leukemia, gastric cancer, and squamous cell lung carcinomas^11–13^. Other small molecule inhibitors of APN, including the natural product curucumin, have been developed and are undergoing testing in the clinic and preclinical models^10^. As with bestatin, specificity has plagued these next-generation compounds due to the limited interactions small molecules can make with the APN pharmacophore. Additional strategies for the therapeutic targeting of APN have utilized tumor-homing peptides based on the NGR motif that bind to APN and deliver cytotoxins to cancer cells^14^. One of these agents, a cyclic version of the NGR peptide complexed to the human tumor necrosis factor alpha, is currently undergoing Phase III clinical trials in mesothelioma^15^.

Understanding substrate specificity is essential to the design of molecules that inhibit the enzymatic activity of APN. Although the preference of APN for neutral amino acid residues at the P1 position has been generally established, little is known about the physical basis for this preference and even less is known about the downstream prime-side specificity of APN. In this study, we performed a comprehensive evaluation of APN substrate specificity and identified key structural features that dictate the specificity of the protease. Using an unbiased mass spectrometry-based peptide library assay, we determined the P1–P4′ substrate preferences of APN and prioritized candidate peptide substrates in the library for rational inhibitor design. Six crystal structures of APN complexed with different amino acids in the P1 position were solved and provided a structural basis for the P1 substrate specificity. From these crystal structures, a peptide was modelled into the specificity pocket to highlight key interactions responsible for dictating the extended prime-side substrate specificity. Using a substrate derived from the peptide library, we developed a novel substrate-based cyclic peptide inhibitor that was specific for APN. Our inhibitor specifically bound to APN-expressing prostate cancer cell lines *in vitro*, decreasing their clonogenic survival, and was an effective therapeutic, leading to decreased tumor growth *in vivo* in xenograft models of prostate cancer.

## Results

### Determination of the substrate specificity of APN

**Figure 1:**
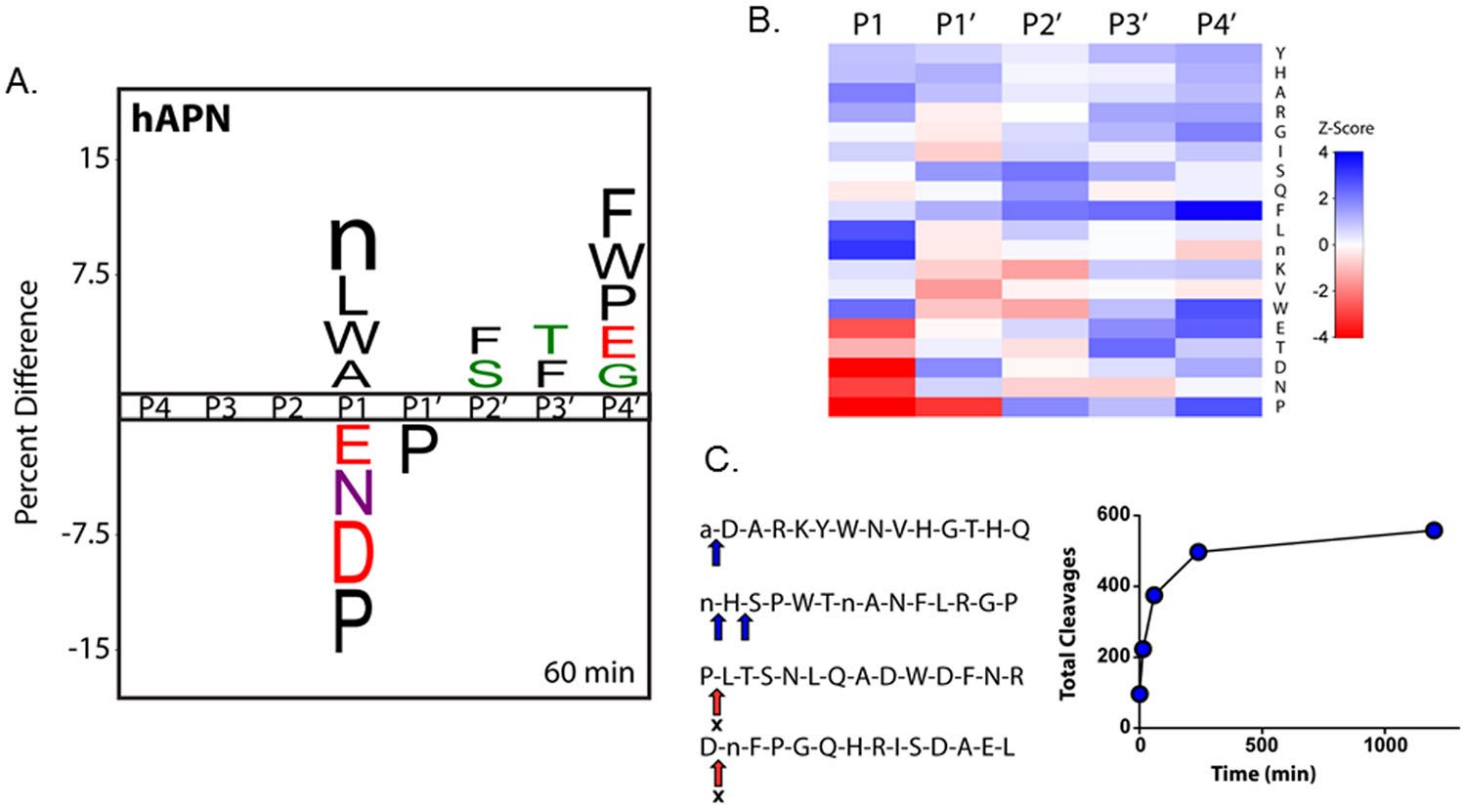
Global identification of human aminopeptidase N (hAPN) substrate specificity with the MSP-MS assay. (**A**) IceLogo representation of P1–P4′ specificity at the 60 min assay time point (*P* ≤ 0.05 for all residues shown; “n” is norleucine). Residues with a positive percent difference are considered favorable at a given position; residues with a negative percent difference are considered disfavorable. (**B**) Heat map representation of hAPN P1–P4′ specificity at the 60 min assay time point calculated using Z-scores at each position. Favored residues are colored blue (Z-score > 0) and disfavored residues are colored red (Z-score < 0). iceLogo representations and heat maps for the 15, 240, and 1200 min assay time points are provided (Supplementary Figure 1). (**C**) Example 14-mer peptides from the MSP-MS library are shown with primary and secondary cleavages indicated with a blue arrow. “X” indicates that no cleavage was detected at the indicated position. A progress curve is provided depicting the total cleavages observed at each assay time point.

To determine the substrate specificity of APN, recombinant human APN (hAPN) was profiled using an unbiased and global substrate profiling approach referred to as Multiplex Substrate Profiling by Mass Spectrometry (MSP-MS)^16^. The MSP-MS assay uses a 228-member library of 14-mer synthetic and unmodified peptide substrates that were rationally designed to maximize physicochemical diversity within a small sequence space^17^. For specificity determination, hAPN was incubated with the MSP-MS peptide library and time-dependent peptide cleavage products were identified with liquid chromatography tandem mass spectrometry (LC-MS/MS). Statistical analysis that considers both cleaved and uncleaved positions in the peptide library^18^ was subsequently performed to construct an iceLogo representation of hAPN P1–P4′ specificity as well as a corresponding heat map based on comprehensive Z-scores for each position (Fig. 1A,B, and Supplemental Figure 1).

In agreement with prior P1 specificity profiling using single-amino acid fluorogenic substrates^5^, hAPN displayed broad specificity at the P1 position. In particular, hAPN exhibited a significant preference for P1 hydrophobic residues (such as norleucine, leucine, tryptophan, and alanine), whereas proline, asparagine, and acidic residues (aspartic acid and glutamic acid) were significantly disfavored. We note that norleucine is used as an isostere for methionine in the MSP-MS library. Inspection of individual peptide cleavage events within the MSP-MS time course supported these overarching P1 specificity preferences with N-terminal cleavages being impaired or blocked by disfavored residues at the P1 (or neo-P1) position (Fig. 1C and Supplemental Figure 2). In addition to providing P1 specificity for hAPN, our global analysis revealed numerous non-prime-side specificity features. In particular, these features include, among others, a significant preference for certain hydrophobic residues (such as tryptophan, phenylalanine, and proline) at the P4′ position, a preference for serine/threonine or phenylalanine at the P2′ and P3′ positions, and a decreased preference for proline at the P1′ position (Fig. 1A,B, and Supplemental Figure 2).

### Structural basis for APN P1 substrate specificity

**Figure 2:**
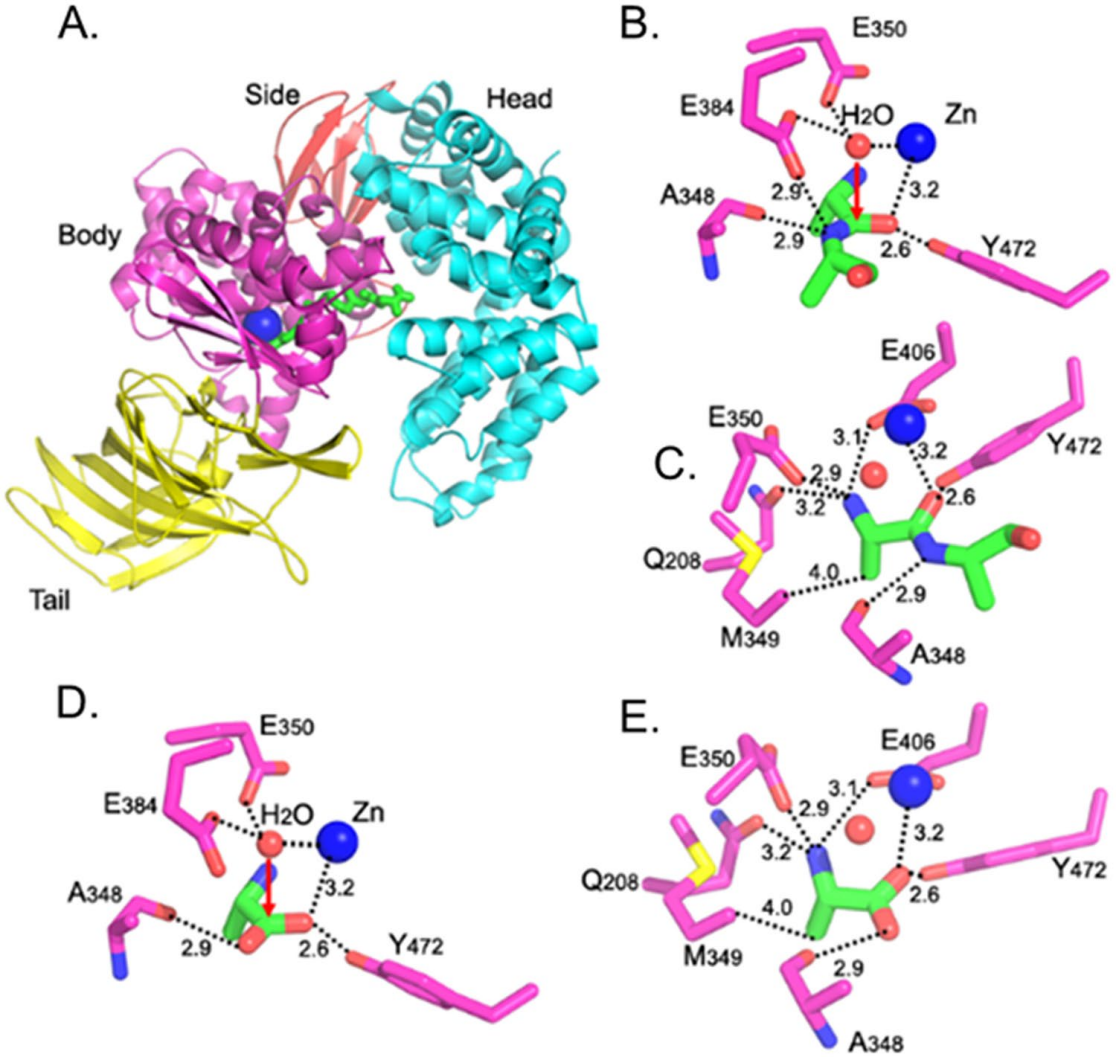
Catalytic mechanism of pAPN. (**A**) Overall structure of pAPN complexed with a peptide substrate (PDB 4FKF). pAPN contains four domains: head (in cyan), side (in brown), body (in magenta), and tail (in yellow). Zinc is shown as a blue ball, and the peptide substrate is in green. (**B**) Interactions between catalytic residues of pAPN (in magenta) and the scissile peptide bond of the peptide substrate (in green). Catalytic water is shown as a red ball. (**C**) Another view of the structure in panel (B) to show all of the interactions between pAPN and the N-terminal residue of the peptide substrate. (**D**) Interactions between catalytic residues of pAPN (in magenta) and product of APN catalysis - free alanine (in green) (PDB 4FKH). (**E**) Another view of the structure in panel (D) to show all the interactions between pAPN and free alanine.

To provide a molecular basis for APN substrate recognition and catalysis, crystal structures were solved of APN bound to natural free amino acids with varied P1 preferences (methionine, leucine, arginine, glycine, isoleucine, and aspartic acid) to probe the influences of the extended binding pocket. Due to the nearly identical architecture of the active sites and P1 specificities of pAPN and hAPN, pAPN was used for analysis because of its propensity to form high quality crystals^5, 6^. The crystal structure of pAPN in complex with the free amino acid alanine and a seven amino acid poly-alanine peptide substrate were initially determined (Figure 2). The seahorse-shaped ectodomain of pAPN contains 4 domains, head, side, body, and tail (Fig. 2A). The N-terminal residue of peptide substrates is firmly anchored in the spacious active site of APN between the head and body domains with residues Gln208, Glu350 and Glu406 forming hydrogen bonds with the free N-terminal amine group (Fig. 2B,C). The nitrogen of the scissile peptide bond forms a hydrogen bond with the electron-repelling carbonyl oxygen of Ala348, while the carbonyl oxygen of the scissile peptide bond interacts with the electron-attracting zinc and Tyr472 (Fig. 2B,C). The resonating electrons of the scissile peptide bond are pulled towards the carbonyl oxygen, thus, destabilizing the bond and making it available to nucleophilic attack by the zinc-activated water molecule. At the same time, the activated nitrogen of the scissile peptide bond is also ready to accept a proton from the catalytic water through the side chain of Glu384. In the presence of free alanine, the carbonyl oxygen of Ala348 from pAPN maintains a hydrogen bond with the carboxyl group of the free alanine, suggesting that the carboxyl group oxygen of free alanine near Ala348 is protonated. Protonation of this carboxyl group oxygen of free alanine is due to simultaneous deprotonation of the other carboxyl group oxygen of free alanine by zinc and Tyr472 (Fig. 2D,E).

**Figure 3:**
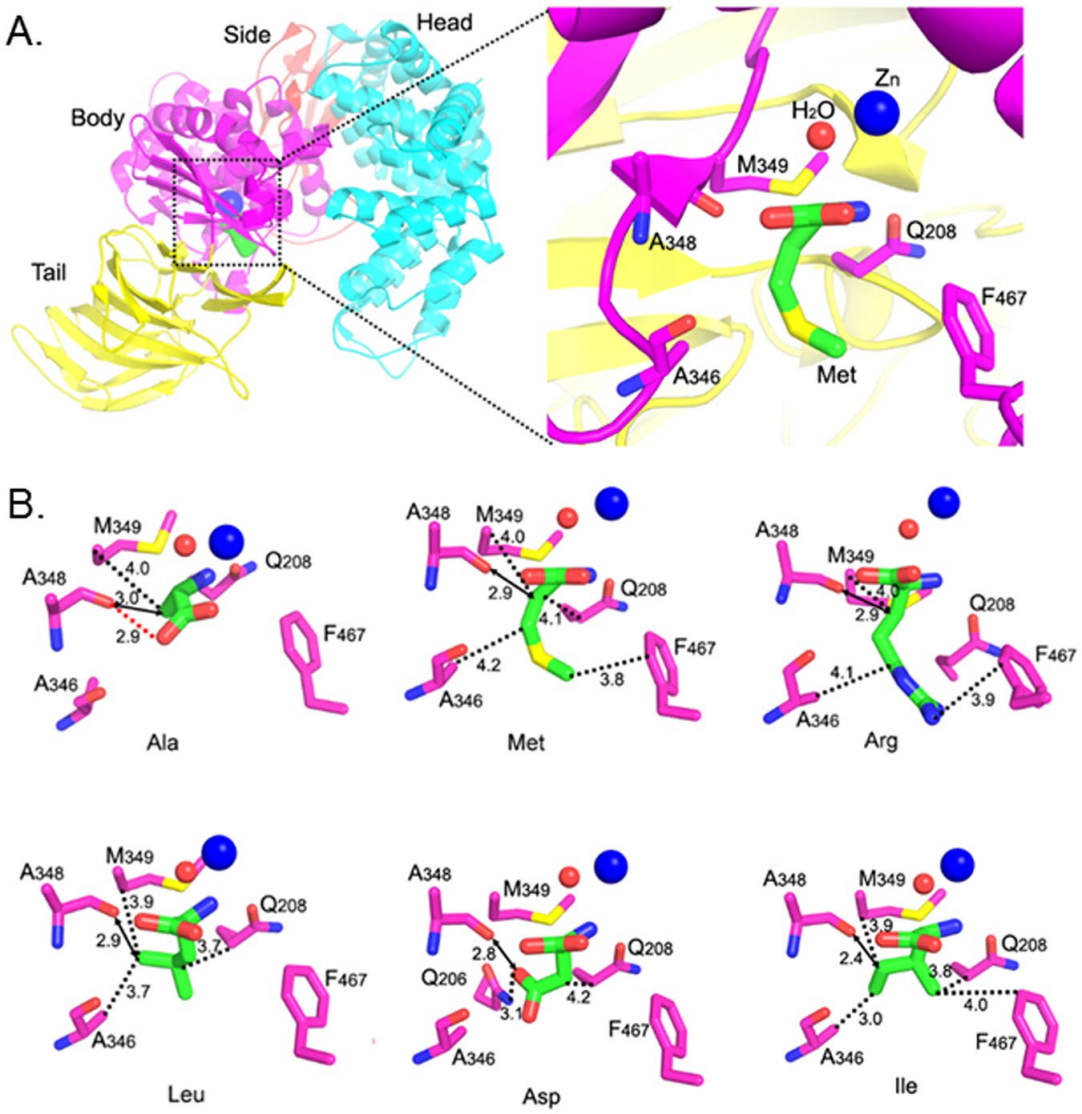
Crystal structures of pAPN complexed with free amino acids. (**A**) The amino acid-binding pocket in pAPN. Left: the overall structure of pAPN complexed with methionine (in green). Right: an enlarged view of the amino acid-binding pocket in the pAPN body domain. The orientation of the view on the right is derived by rotating the view on the left 90° clockwise along a vertical axis. (**B**) Crystal structures of pAPN complexed with amino acids that are favored (Ala, Met, Arg and Leu) and disfavored (Ile and Asp) P1 residues for APN. pAPN residues are in magenta, and amino acids are in green. The catalytically critical hydrogen bond between the carbonyl oxygen of Ala348 and the C-terminal carboxyl group of amino acids is shown as a red dashed line in the structure with Ala, but is omitted in other panels for clarity. The interactions between the carbonyl oxygen of Ala348 and amino acid side chains are shown as black dashed lines. The distance between the carbonyl oxygen of Ala348 and the nearest atom on amino acid side chains is shown as a bidirectional arrow.

Because pAPN forms the same interactions with a free alanine and the N-terminal alanine residue of a poly-alanine peptide substrate, we believe that the crystal structures of pAPN complexed with free amino acids reflect the interactions between pAPN and the N-terminal amino acid residues of at least simple peptide substrates^4^. All of the free amino acids in the crystal structures bound to pAPN in a similar manner as alanine, with the exception of key differences in their side chain interactions. The amino acid side chains were oriented in a pocket in the pAPN body domain with hydrophobic walls and an open end (Fig. 3A). The side chains with different lengths were able to fit into the pocket because of the open end and were found to primarily form hydrophobic interactions with the walls of the pocket (Fig. 3B). For example, C_β_, C_γ_, and C_ε_ of the methionine side chain formed hydrophobic interactions with Met349/Gln208, Ala346, and Phe467 on the pocket walls (Fig. 3B). Polar side chains were also able to form additional interactions with pAPN as in the case of arginine where the guanidine group formed a cationic-Pi interaction with Phe467 (Fig. 3B). In general, the APN-binding affinity of amino acids is positively associated with the extent of hydrophobic or other affinity-increasing interactions between their side chains and the pocket walls. Consequently, amino acids with long, nonpolar side chains like leucine generally have high APN-binding affinity. Analysis of the crystal structures of pAPN complexed with non-favored amino acids found that those residues all shared one unique feature – their side chains had unfavorable interactions with the catalytically critical carbonyl oxygen of Ala348 from pAPN (Fig. 3B). For example, the C_γ_ atom of isoleucine was 2.4 Å away from the carbonyl oxygen of Ala348, respectively (Fig. 3B). At these short distances, a strong van der waals (VDW) repulsion existed between the atoms. Additionally, the O_δ_ group of aspartic acid formed an unfavorable charge repulsion with the carbonyl oxygen of Ala348 (Fig. 3B). As a comparison, the side chains of amino acids that are good APN substrates form weak or no interactions with the carbonyl oxygen of Ala348 (Fig. 3B).

### The Development of Substrate-Based Inhibitors of APN

To identify sequences for inhibitor development, a selection of 14-mer peptides from the MSP-MS library that underwent complete hydrolysis during the assay were compared using label-free quantitation of peptide cleavage kinetics (Supplemental Figure 2). Five-residue long peptides containing P1–P4′ residues from primary cleavages in the library were synthesized and tested for their ability to prevent the cleavage of a fluorogenic alanine substrate by hAPN. From these initial peptides, only nHSPW was able to appreciably inhibit substrate cleavage with an IC_50_ of 6.5 μM, whereas the remaining peptides all had IC_50_ values above 500 μM (Table 1). Replacement of the P1 residue norleucine of nHSPW with either leucine or alanine did not have a significant impact on the IC_50_, resulting in low μM inhibitors. Complete removal of the P1 norleucine, resulting in the 4-mer peptide HSPW, only decreased the potency of the inhibitor to 43 μM.

**Table 1:** List of Aminopeptidase N inhibitor peptides.

Peptide	IC_50_ (µM)	Peptide	K_i_ (µM)
**Bestatin**	**3**.**7 ± 0**.**4**	cyc-LHSPW	24.7 ± 1.4
nHSPW	6.5 ± 0.5	cyc-LHSP	121.4 ± 21.4
AHSPW	9.4 ± 1.1	cyc-LHS	>500
LHSPW	10.6 ± 1.2	cyc-PHSPW	>500
HSPW	43.3 ± 3.9	cyc-AHSPW	>500
PHSPW	82.5 ± 9.3	cyc-nHSPW	>500
EHSPW	115.4 ± 17.2		
nDQIY	>500	For-HSPW	>500
LDSTF	>500	Suc-HSPW	>500
DSTF	>500	Cbz-HSPW	>500
ADARK	>500	Ac-HSPW	>500
DARK	>500	For-LHSPW	>500
INDFL	>500	Suc-LHSPW	>500
AHLFN	>500	Cbz-LHSPW	>500
FSLSK	>500	Ac-LHSPW	>500
SKSGQ	>500		
AHSTF	>500	cyc-LDSTF	>500
FYLRE	>500	cyc-ADARK	>500
FDWWG	>500		
RDLVD	>500		
EPKVA	>500		

**Figure 4:**
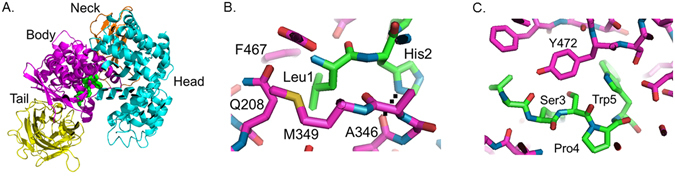
Molecular modeling of the peptide LHSPW in the active site of pAPN. (**A**) Structure of pAPN complexed with the modeled LHSPW peptide (green) depicting the pAPN ectodomain and its four components: head (cyan), neck (brown), body (in magenta), and tail (in yellow). For the modeling of peptide LHSPW in the active site of APN, two crystal structures were used: (**B**) the first three residues (Leu1-His2-Ser3) were modeled from the structure of pAPN complexed with polyalanines (PDB ID: 4NAQ), and (**C**) last two residues (Pro4-Trp5) were from the structure of pAPN complexed with substance P, which also contains a proline at the 4th position (PDB ID: 4HOM).

To define the key prime-side interactions accounting for the inhibition of these HSPW-containing sequences, the peptide LHSPW was modeled into our pAPN crystal structure (Fig. 4A). For modeling purposes, the P1 residue was selected as leucine since we had solved a crystal structure with that residue in the P1 position – we were unable to get a crystal structure of norleucine – and leucine was the second most favored P1 residue in our MSP-MS assay. The first three residues (Leu1-His2-Ser3) were modeled from a poly-alanine structure and last two residues (Pro4-Trp5) were from the structure of pAPN complexed with substance P, which also contains a proline at the 4th position (PDB ID: 4HOM). As predicted from the P1 structural data, the side chain of Leu1 in both peptides points into a hydrophobic pocket surrounded by APN residues M349, Q208, and F467 (Fig. 4B). The side chain of His2 forms a hydrogen bond with the main chain carbonyl oxygen of pAPN residue A346. Pro4 forms a turn in the peptide, whereas the side chains of Ser3 and Trp5 both form VDW interactions with APN residue Y472 (Fig. 4C).

Using the HSPW core sequence, we next sought to create true inhibitors of APN that were not cleavable substrates and with the potential proteolytic stability required for *in vivo* studies. Addition of N-terminal capping groups to the peptides completely abrogated any inhibitory ability. Next, the peptides were cyclized through the formation of a disulfide bond using N and C-terminal cysteine residues (Cys-XHSPW-Cys = cyc-XHSPW). The cyclization of the parent nHSPW peptide through cysteine residues (cyc-nHSPW) also eliminated any measureable inhibition of APN; however, cyclization of the leucine derivative (cyc-LHSPW) yielded an inhibitor with a K_i_ of 24.7 μM. Truncation of the core sequence revealed the importance of Pro4 and Trp5 in determining the affinity of the cyclic peptide, resulting in K_i_ values of 121.4 μM and >500 μM for the cyclic tetrapeptide and tripeptide derivatives, respectively.

### Biological Evaluation of cyc-LHSPW

**Figure 5:**
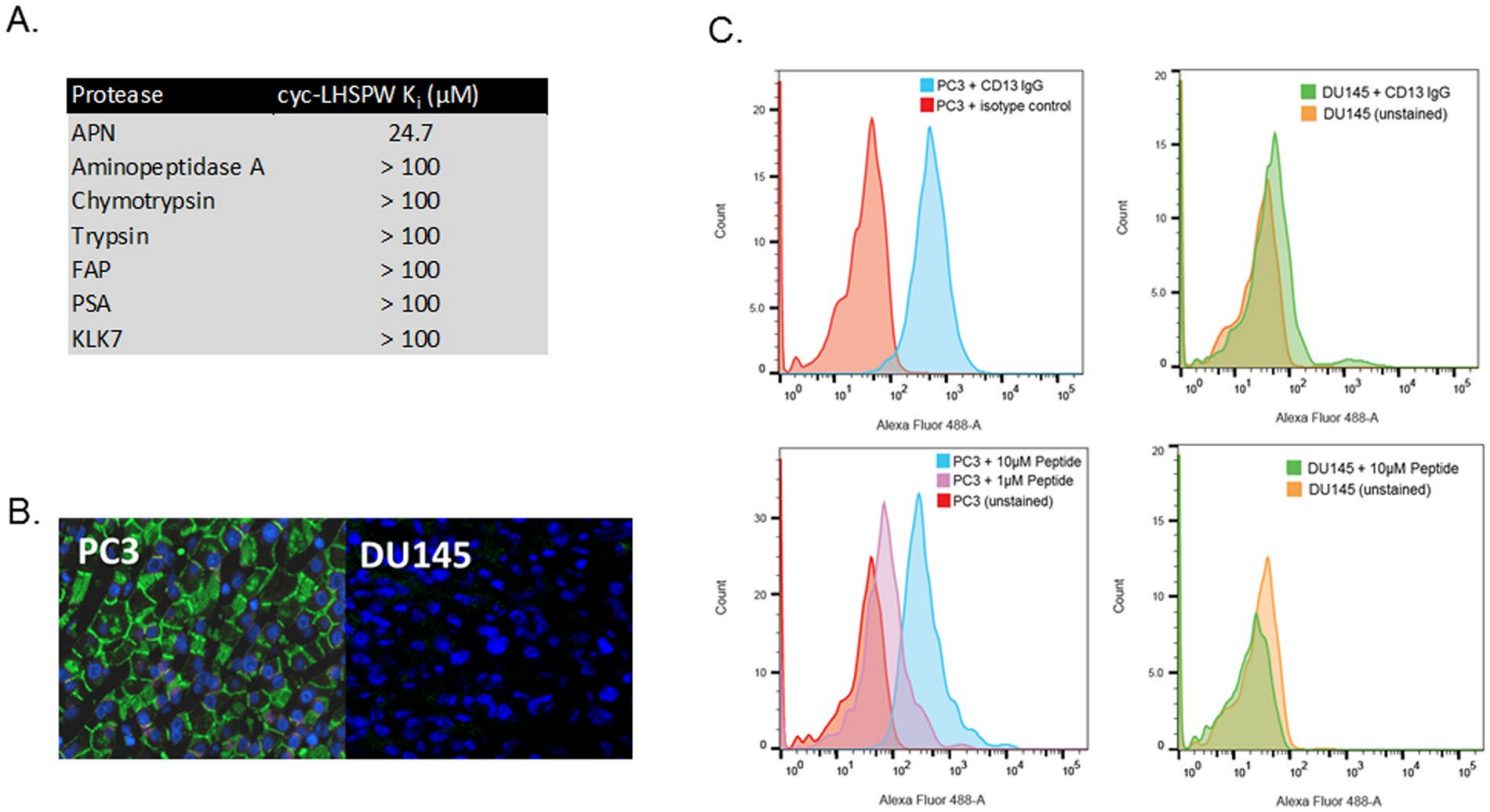
Determining the specificity of cyc-LHSPW for APN. (**A**) The cyc-LHSPW peptide was specific inhibitor of APN when compared to a panel of proteases. (**B**) Fluorescence microscopy of frozen PC3 (left) and DU145 (right) xenograft sections with cyc-LHSPW-(Gly)_4_-FITC. Sections were incubated with 250 nM of cyc-LHSPW-(Gly)_4_-FITC overnight and then visualized. The merged fluoresence channels are cyc-LHSPW-(Gly)_4_-FITC (green) and nuclei (DAPI, blue). (**C**) Analysis of APN inhibitor binding to PC3 (left) and DU145 cells (right) by flow cytometry. The peptide (cyc-LHSPW-(Gly)_4_-FITC) selectively labeled the APN-expressing PC3 cells over the APN-null DU145 cells. The cells surface expression of APN was by confirmed by staining both cells with a FITC conjugated anti-CD13 (APN) antibody from Miltentyi.

Next, the specificity of the lead peptide inhibitor cyc-LHSPW was tested against a panel of cancer-associated proteases with wide-ranging substrate specificities (Fig. 5A). APN was the only protease inhibited by cyc-LHSPW with chymotrypsin, trypsin, PSA, KLK7, aminopeptidase A, and the exopeptidase FAP all having K_i_ values above 100 μM. Having assessed selectivity for purified APN, we next evaluated the ability of cyc-LHSPW to preferentially bind to cells that express surface APN using flow cytometry (Fig. 5B). For flow cytometry, we synthesized a fluorophore-containing version of the peptide possessing a (Gly)_4_-FITC group coupled to the C-terminal cysteine (Supplemental Figure 4). The expression of cell surface-associated APN was examined in the androgen receptor negative neuroendocrine prostate cancer cell lines PC3 and DU145 using a commercially available antibody. The cell line PC3 was found to express surface-associated APN, whereas no detectable expression was found for the DU145 cell line by this method. When incubated with both cells lines, the FITC labeled cyc-LHSPW peptide preferentially bound to PC3 cells with no noticeable labelling of DU145 cells. The peptide was next tested on frozen PC3 and DU145 tumor xenografts sections. Preferential labeling of the PC3 xenograft section was evident, furthering attesting to the specificity of cyc-LHSPW by fluorescence microscopy.

**Figure 6:**
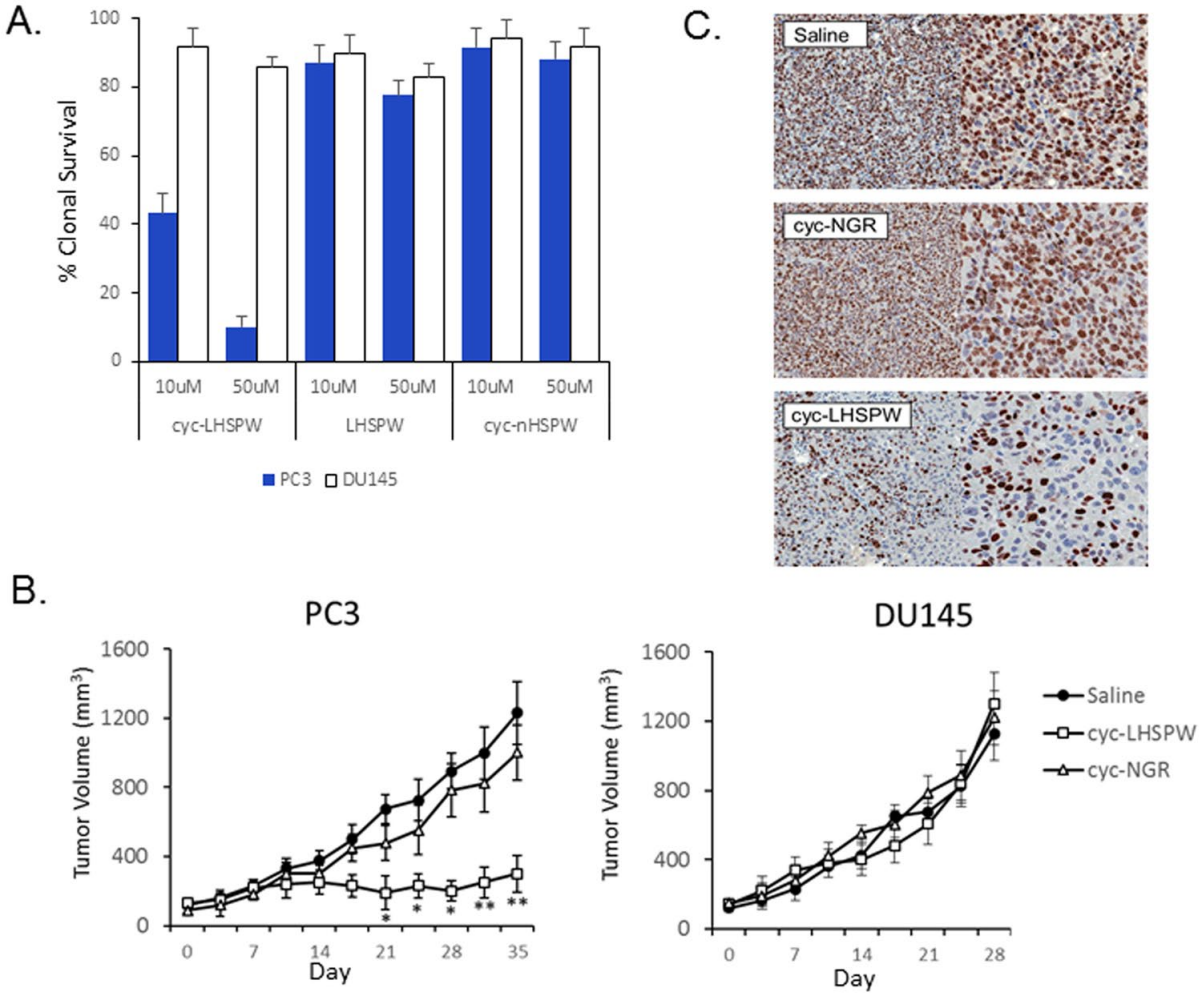
The therapeutic evaluation of cyc-LHSPW *in vivo* and *in vitro*. (**A**) The effect of the three peptides cyc-LHSPW, LHSPW and cyc-nHSPW on the clonogenic survival of APN-expressing PC3 cells and APN-null DU145 cells. (**B**) Tumor growth of PC3 and DU145 xenograft mice treated with 40 mg/kg of cyc-LHSPW, cyc-NGR or saline control three times a week for four weeks. Mice were first injected with drug via tail vein starting at day 0 of the study and continued until tumor volumes >1000 mm^3^ were observed as dictated by our animal protocol. Each treatment group consisted of n = 9 mice/xenograft. Statistical significance of cyc-LHSPW compared with control is denoted by: *P < 0.05; and **P < 0.01, as determined by the Student t test. (**C**) At the end of the study, PC3 tumors were removed from the treated arms and stained for the proliferation maker Ki67. Decreased Ki67 staining is evident in the cyc-LHSPW treated arm.

The potential therapeutic efficacy of cyc-LHSPW was next tested *in vitro* using the PC3 and DU145 cell lines. The APN inhibitor demonstrated a specific and pronounced therapeutic benefit resulting in the decreased clonal survival of PC3 cells. No significant biological effect was observed on either cell line after treatment with the hydrolyzable LHSPW peptide or the cyc-nHSPW derivative. Prior to *in vivo* therapeutic efficacy studies, a toxicity study was performed with the i.v. administration of cyc-LHSPW. We found that a single high dose of 100 mg/kg was well tolerated by the mice; however, multiple doses at this concentration, as would be needed, resulted in cachexia. It was found that a dose of 40 mg/kg administered three time a week for four weeks (t.i.w. x4) was well tolerated, i.e. no signs of morbidity. Mice bearing established PC3 and DU145 xenografts were treated systemically via tail vein injection with 40 mg/kg of the tumor homing APN peptide cyc-NGR or the cyc-LHSPW peptide t.i.w. x4 (Fig. 6B). Inhibition of tumor growth in the PC3 xenograft mice treated with cyc-LHSPW was significant (P < 0.05) three weeks into the trial when compared to the saline- and cyc-NGR-treated arms. This therapeutic effect persisted to the end of the study with the cyc-LHSPW-treated mice having a tumor volume of 309.3 ± 92.7 mm^3^ at day 35. Little therapeutic effect was observed in the cyc-NGR-treated mice with both the saline- and cyc-NGR-treated arms having tumor volumes >1000 mm^3^ at the end of the study. PC3 tumors were removed and stained for the cellular proliferation marker Ki67. Both the saline- and cyc-NGR-treated tumors had profoundly higher populations of K_i_67 positive cells compared to the cyc-LHSPW-treated tumors (Fig. 6C). No therapeutic effect observed in the APN-negative DU145 tumors treated with either cyc-LHSPW or cyc-NGR.

## Discussion

The proteases responsible for the increased proteolysis associated with aggressive forms of cancer represent candidate therapeutic targets. The over-expression of APN on the surface and neo-vasculature of solid tumors has made it the most studied member of the M1 family of metalloproteases and the most targeted. A number of groups have developed molecules targeting APN ranging from monoclonal antibodies to synthetic peptidomimetic inhibitors of its enzymatic activity^19–24^. Perhaps the most well-known for APN is the cyclic tumor-homing peptide asparagine-glycine-arginine (cyc-NGR) originally discovered through *in vivo* peptide phage display^25^. This peptide has been radiolabeled for nuclear imaging and has been used to deliver therapeutic peptides and proteins *in vivo* to APN-expressing tissues^26–28^. The NGR motif mimics the structure of extracellular matrix proteins that APN is known to bind but not degrade. There are a number of drawbacks associated with the use of the cyc-NGR peptide, foremost among them is deamidation of the asparagine residue^25^. This spontaneous reaction, enhanced when the asparagine is adjacent to a glycine residue, results in the formation of isoaspartate-glycine-arginine (isoDGR) which is a ligand capable of binding to α_V_β_3_ integrin and several other integrins with lower affinity. This deamidation can lead to decreased specificity and potential off target affects^29^. Additionally, as further demonstrated by our study, the cyclic-NGR peptide alone is not therapeutic, rather it is only therapeutic when conjugated to toxins. Thus, there is a great need for low-molecular weight APN-targeted therapeutics that are stable and specific.

The development of targeted agents for APN has been greatly hindered by two factors – the incomplete understanding of APN substrate specificity and, until recently, the paucity of high-resolution atomic structures. Here, we have presented the most comprehensive examination of the substrate specificity of APN to date and detailed the key structural interactions that dictate this specificity. By understanding the molecular architecture of APN at the atomic level and investing its specificity with our global profiling technique, we were able to rationally design peptide inhibitors of its enzymatic activity. The S1 pocket of APN has hydrophobic walls with an open end resulting from the presence of Met349, Phe467 and Tyr472. As predicted from the S1 architecture, the P1 specificity of APN favored hydrophobic residues. Amino acids with large positively charged side chains (such as arginine and lysine) are also tolerated as substrates because their side chains can form hydrophobic and polar interactions with aforementioned residues in the S1 pocket. In contrast, acidic residues (glutamic acid and aspartic acid) and short-branched residues (such as asparagine and proline) are strongly disfavored in the P1 position due to charge and VDW repulsion, respectively.

The active site of APN is expansive with openings on three sides allowing for the accommodation of large peptide substrates. Its exclusive mono-aminopeptidase activity is the direct result of hydrogen bonds formed between active site residues Gln208, Glu350 and Glu406 with the N-terminal amine group of the peptide substrate. Di-aminopeptidase activity is not observed because favorable hydrogen bonds cannot be made to anchor the peptide in the proper orientation for bond hydrolysis. The importance of these hydrogen bonds in peptide binding was also evident in our inhibitor study in which the N-termini of HSPW peptides were capped with different groups. By removing one or more potential hydrogen bonds from the N-terminus, the capped peptides were unable to bind, resulting in poor inhibition. In addition to these P1 residues, our global analysis revealed previously uncharacterized prime-side specificity features that were dictated by hydrophobic interactions in addition to hydrogen bonding with main chain groups. These important interactions were evident when the HSPW core sequence of our lead inhibitor was truncated. The removal of Trp5 or both Pro4 and Trp5 abrogated the ability of the peptide to inhibit APN. The critical interactions these two residues make with the active site were observed in our modelling experiment documenting that both residues form VDW interactions with Y472. The end result of our study was a cyclic peptide, cyc-LHSPW that was a potent and specific inhibitor of APN. By inhibiting the enzymatic activity of APN, we found that cyc-LHSPW was therapeutic both *in vitro* and *in vivo* in a model of highly aggressive neuroendocrine prostate cancer. With this aggressive form of cancer on the rise, it is highly plausible the future treatments for neuroendocrine prostate cancer could involve targeting APN^30^.

Our investigation into the substrate specificity of APN underscored the utility of the MSP-MS assay over conventional techniques for determining aminopeptidase specificity. A number of limitations exist when using single-amino acid chromogenic or fluorogenic substrates to determine aminopeptidase specificity with P1′ reporter groups, as an example, potentially altering the P1 specificity profile. In addition to reporting on prime-side specificity, our global approach also allowed for the identification of individual synthetic peptides that are bona fide substrates and served as a template for APN inhibitor design. APN has emerged as important pericellular protease target in aggressive cancer. We envision that the substrate specificity preferences identified here will both guide the development of next-generation APN-targeted therapeutics as well as aid in the discovery of its endogenous substrates to further expand our knowledge of its role in biology.

## Materials and Methods

### APN expression and purification

Porcine APN ectodomain (residues 62–963) and human APN ecdomain (residues 66–967) were expressed and purified as previously described^31^. Briefly, APN ectodomains containing N-terminal honeybee-melittin signal peptide and C-terminal His_6_ tag were expressed in insect cells, secreted to cell culture medium, and purified sequentially on Ni-NTA column and gel-filtration column.

### Multiplex Substrate Profiling by Mass Spectrometry (MSP-MS)

MSP-MS assays were carried out as described previously^16^. Briefly, 0.2 µg/mL recombinant human APN from R&D Systems (catalog #: 3815-ZN-010) and matched no-enzyme control were assayed against a diverse library of 228 tetradecapeptides pooled at 500 nM in D-PBS (pH 7.4) containing 1 mM TCEP. After 1, 15, 60, 240, and 1200 min, 30 µL of assay mixture was removed, quenched with 7.5 µL 20% formic acid, and flash-frozen in liquid N_2_. Prior to mass spectrometry acquisition, peptide samples were desalted using C_18_ desalting tips and rehydrated in 0.2% formic acid. LC-MS/MS data were acquired using a Thermo Scientific LTQ-FT mass spectrometer, which was equipped with a Thermo Scientific EASY-Spray Ion Source, EASY-Spray PepMap C_18_ Column (3 µM, 100 Å), and Waters nanoACQUITY UPLC System.

Peptide peak lists were generated using MSConvert from the ProteoWizard Toolkit^32^, and data were searched against the 228-member peptide library using Protein Prospector software (v.5.17.0, University of California, San Francisco)^33^. Protein Prospector score thresholds were selected with a minimum protein score of 15 and minimum peptide score of 10. Maximum expectation values of 0.01 and 0.05 were used for protein and peptide matches, respectively. Substrate specificity profiles were generated with iceLogo software^18^ using all possible cleavages in the MSP-MS library (n = 2,964) as the negative data set as described^16^. MS1 extracted ion chromatograms for label-free quantitation of select substrate and product species were generated using Skyline software (v.3.5, University of Washington)^34^. Specificity constants (*k*_cat_/*K*_m_) were calculated as described^16^. All raw spectrum (.RAW) files from MSP-MS experiments in this study are available at the ProteoSafe resource (ftp://MSV000080171@massive.ucsd.edu/; username MSV000080171, password: hAPN).

### Structure determinations of porcine APN/amino acid complexes

Porcine APN ectodomain was crystallized as previously described^35^. Briefly, pAPN at 10 mg/mL concentration in buffer containing 20 mM Tris pH7.2 and 200 mM NaCl was crystallized by itself and then amino acid was soaked into the pAPN crystal. Crystallization of pAPN was set up with 1 µL protein solution and 1 µL well solution containing 18% PEG3350, 200 mM Li_2_SO4, and 100 mM HEPES pH7.2 mixed together in sitting drops. Crystals of pAPN were grown at 4 °C for two weeks, and were then transferred to amino acid-soaking solution containing 5 mM amino acid, 20% ethylene glycol, 25% PEG3350, 200 mM Li_2_SO4, and 100 mM HEPES pH7.2 for another two days. The crystals were flash frozen in liquid N2 and used for data collection. Data were collected at APS beamline 24-ID and ALS beamline 4.2.2. X-ray diffraction data were processed using HKL2000^36^. The structures of pAPN complexed different amino acids were determined by directly refining the model of the unliganded pAPN structure (PDB 4FKE) against the amino acid-soaked crystal data. Programs CNS^37^ and CCP4 refmac^38^ were used for structure refinement. Program COOT was used for model building and structural superposition^39^.

The six amino acids were soaked into pAPN crystals to determine the structures of the pAPN/amino acid complexes. The structure of the unliganded pAPN was used to determine the structures of amino acid-soaked pAPN. Objective Fo-Fc omit maps were calculated in the absence of amino acids and showed strong electron density for each of the amino acids (Supplemental Figure 4). Based on these maps, the models of amino acids were built, and the models of pAPN/amino acid complexes were further refined. Crystallographic statistics can be found in Supplemental Figure 5.

### Peptide synthesis and inhibitor testing

The peptides used in this study were custom synthesized by AAPPTEC of Louisville, KY using traditional Fmoc solid-phase peptide synthesis^40^. All of the peptides were purified and characterized by AAPPTEC. The peptides used in all of the studies were greater than >95% purity as determined by high-performance liquid chromatography. Inhibition assays were performed in triplicate in a 100 mM Tris pH 7.2, Brij 35 0.0002% w/v assay buffer at 37 °C with 0.1 μM APN and 100 μM H-Ala-AMC (fluorophore). Values were recorded by Infinite pro plate reader every 2 minutes for 30 minutes with laser parameters set for excitation at 355 nm and emission at 460 nm. As the inhibition of APN was competitive, the values for the inhibition constant, K_i_, were determined from the IC50 using the Cheng-Prusoff equation: K_i_ = IC_50_/(1 + [S]/Km).

### Flow cytometry and microscopy

DU145 and PC3 cells were washed with PBS and harvested mechanically with a cell scraper. A total of 1 × 10^6^ cells were incubated with 500 nmol/L cyc-LHSPW-(Gly)_4_-FITC or anti-CD13 antibody (22A5-FITC, Miltenyi) for 60 minutes at 4 °C. Stained samples and controls were assayed on a BD Facscalibur. Tumor sections frozen in OCT medium were sectioned at a thickness of 7 μm and briefly fixed in cold acetone for 10 min. After rinsing in PBS, the sections were stained with 250 nM cyc-LHSPW-(Gly)_4_-FITC overnight at 4 °C. The sections were washed with PBS and mounted with DAPI Prolong Gold. The slides were visualized using a Nikon 6D High Throughput Epifluorescence Microscope.

### *In vitro* and *in vivo* therapeutic studies

The clonogenic survival assays were conducted as previously described^41^. Mouse care and treatment was approved by and performed in accordance with the guidelines of the University of Minnesota Institutional Animal Care and Use Committee. Cells maintained under standard conditions were detached by treatment with 0.25% trypsin–EDTA solution and washed in Hank’s balanced salt solution (HBSS). They were then suspended in a 60% mixture of Matrigel Matrix (BD Biosciences) in HBSS at a concentration of 1.0 × 10^6^ cells per 100 μL of solution. The cells were then injected into the subcutis overlying the rear flanks of 6-week-old male nude mice (Harlan). Once the tumors were established, the animals were randomized into three treatment groups per xenograft. The both cyc-LHSPW and cyc-NGR were dissolved in DMSO and diluted in sterile PBS to a final DMSO concentration of 5% v/v. The mice were then dosed via tail vein injection with 40 mg/kg of the cyclic peptides or saline at days three times a week for four weeks total. Tumor measurements were made twice weekly with calipers and the tumor volume (in mm^3^) was calculated by the formula 0.5236 × length (L) × width (W) × height (H). The endpoint of the study was either five weeks after the first treatment dose or when the tumors reached a volume of 1,000 mm^3^ as dictated by our animal protocol. PC3 tumors were removed from euthanized animals, formalin fixed and stained for Ki67 using the manufacturer’s protocol.

### Statistical analysis

Data were analyzed using the unpaired, two-tailed Student t test. Differences at the 95% confidence level (P < 0.05) were considered to be statistically significant.

## Electronic supplementary material

Supplementary Information
