# Supplementary material for: The Rational Design of Therapeutic Peptides for Aminopeptidase N using a Substrate-Based Approach
Source: Sci Rep. 2017 May 2;7:1424. doi: 10.1038/s41598-017-01542-5 (PMC5431086; doi:10.1038/s41598-017-01542-5)
Supplement: Supplementary file 1 — Supplementary Information [file 41598_2017_1542_MOESM1_ESM.pdf]

Supplemental Information

The Rational Design of Therapeutic Peptides for Aminopeptidase N using a Substrate-Based Approach

Shilvi Joshi[1], Lang Chen [1], Michael B. Winter[2], Yi-Lun Lin[1], Yang Yang[1], Mariya Shapovalova[1], Paige M. Smith[1], Chang Liu [1], Fang Li[1] and Aaron M. LeBeau[1]

[1] Department of Pharmacology, University of Minnesota Medical School, Minneapolis, MN 55455, USA
[2] Department of Pharmaceutical Chemistry, University of California, San Francisco, CA 94153, USA

Supplemental Figure 1

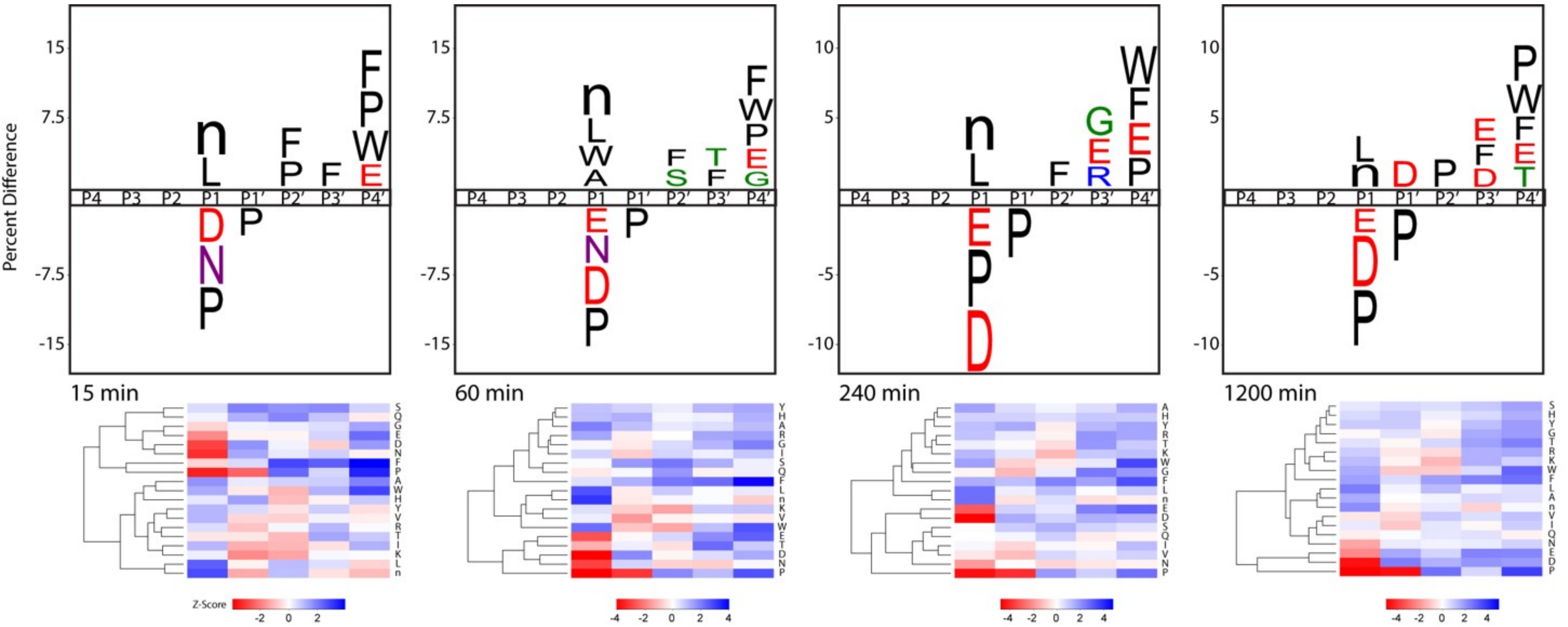

Supplemental Figure 1: Global identification of hAPN substrate specificity with the MSP-MS assay. (Top) iceLogo representation of P1-P4' specificity at the 15, 60, 240, and 1200 min assay time points ($P \leq 0.05$ for all residues shown; "n" is norleucine). (Bottom) Heat map representations of hAPN P1-P4' specificity at the 15, 60, 240, and 1200 min assay time points calculated using Z-scores at each position. Favored residues are colored blue (Z-score > 0) and disfavored residues are colored red (Z-score < 0).

Supplemental Figure 2

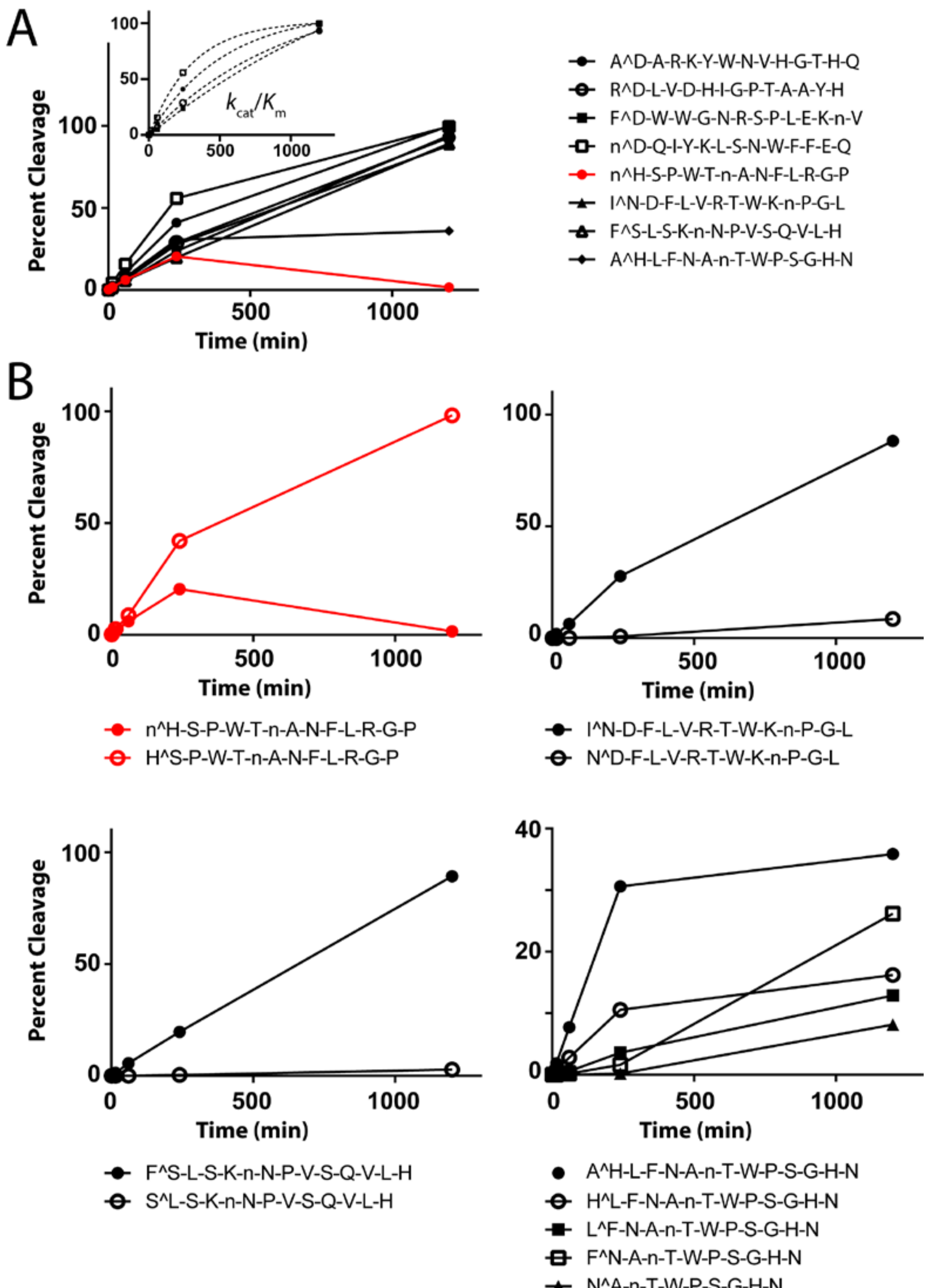

Supplemental Figure 2: Comparison of hAPN cleavage against a panel of 14-mer peptides from the MSP-MS library that underwent complete hydrolysis during the assay time course and that were selected for inhibitor development (Table I). A.) Cleavage time courses for hAPN removal of the primary N-terminal amino acid in the panel of substrates. Inset.) Determination of k_{cat}/K_m values for substrates that did not undergo secondary cleavage events. Notably, these substrates contain aspartic acid, which is a disfavorable P1 residue for hAPN, at the second position. Arrows (^) indicate the site(s) of cleavage. k_{cat}/K_m values were as follows: 1.67 (± 0.08) x 10^4, 9.03 (± 0.36) x 10^3, 4.46 (± 0.75) x 10^3, and 2.85 (± 0.15) x 10^4 $M^{-1}s^{-1}$ for A^D-A-R-K-Y-W-N-V-H-G-T-H-Q, R^D-L-V-D-H-I-G-P-T-A-A-Y-H, F^D-W-W-G-N-R-S-P-L-E-K-n-V, and n^D-Q-I-Y-K-L-S-N-W-F-F-E-Q, respectively. B.) Time courses showing secondary cleavage events for a subset of the panel. The five terminal residues (P1-P4’) of the substrate n-H-S-P-W-T-n-A-N-F-L-R-G-P (red) was the most effective hAPN inhibitor in the absence of cyclization (Table I).

Supplemental Figure 3

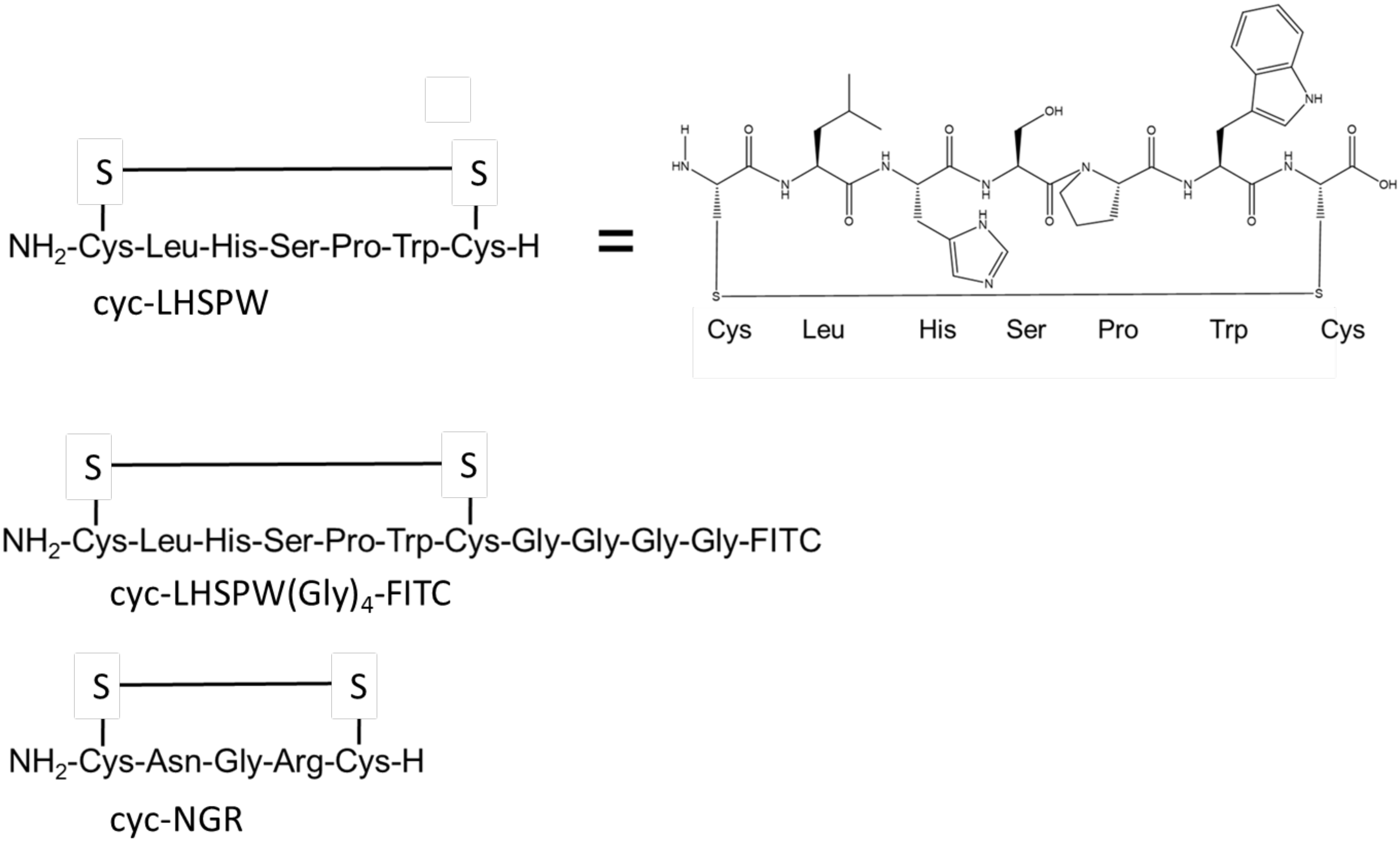

Supplemental Figure 3: Structures of the salient cyclic peptides used in this study.

S

Supplemental Figure 4

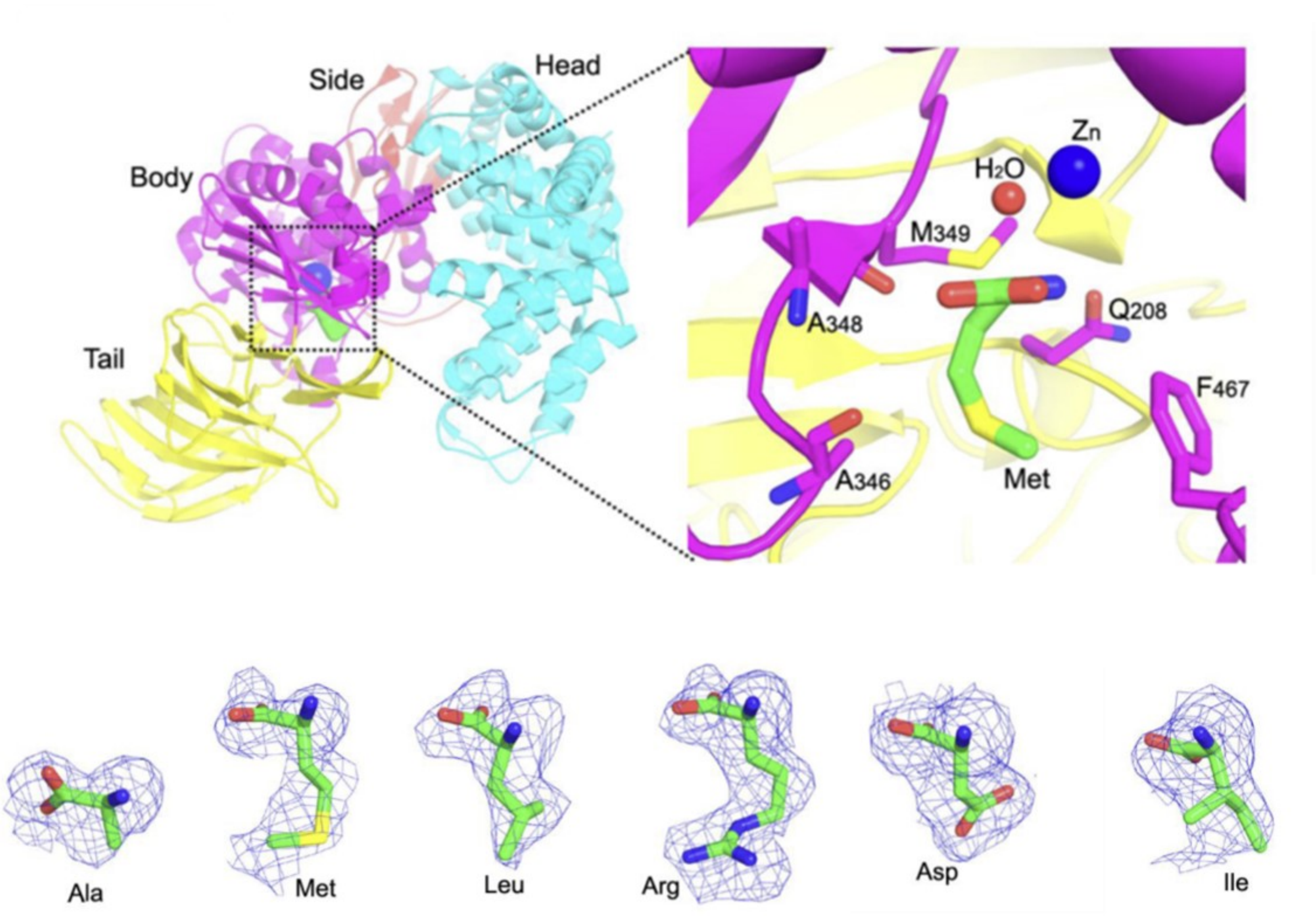

Supplemental Figure 4: Electron density of the amino acid side chains.

Supplemental Figure 5

Data	Arg/pAPN complex	Asp/pAPN complex	Ala/pAPN complex	Ile/pAPN complex	Leu/pAPN complex	Met/pAPN complex
PDB code	4IYV	4IYW	4IYY	4IYZ	4IZ1	4IZ2
**Data collection**						
Space group	C2	C2	C2	C2	C2	C2
Cell parameter						
a(Å)	260.7	259.8	260.6	260.4	259.6	260.7
b(Å)	62.9	62.7	62.7	62.9	63.0	62.9
c (Å)	81.7	81.7	81.6	81.7	81.6	81.6
α(°)	90.0	90.0	90.0	90.0	90.0	90.0
β(°)	100.6	100.4	100.3	100.5	100.4	100.4
γ (°)	90.0	90.0	90.0	90.0	90.0	90.0
Wavelength (Å)	1.00003	0.97918	0.97918	1.00003	1.00003	1.00003
Resolution (Å)	50 - 1.86	50 - 2.00	50 - 2.10	50 - 2.15	50 - 2.20	50 - 2.03
No. reflections	107699	85779	75984	69735	65231	84218
% completeness	98.2	97.8	99.9	99.0	99.1	98.9
(last shell)	(84.4)	(97.2)	(99.8)	(92.6)	(95.7)	(90.0)
Rmerge	0.075	0.062	0.061	0.116	0.092	0.085
(last shell)	(0.484)	(0.574)	(0.464)	(0.572)	(0.601)	(0.425)
I/ σ	18.6	19.6	20.1	16.3	22.8	21.4
(last shell)	(1.9)	(2.4)	(3.1)	(2.3)	(2.6)	(3.6)
Redundancy	4.3	3.7	3.7	6.5	7.1	7.4
(last shell)	(2.4)	(3.7)	(3.8)	(4.4)	(4.8)	(5.5)
**Refinement**						
Resolution (Å)	50 - 1.86	50 - 2.00	50 - 2.10	50 - 2.15	50 - 2.20	50 - 2.03
Rwork / Rfree	0.154/0.229	0.140/0.223	0.135/0.213	0.143/0.231	0.142/0.245	0.141/0.216
No. Atom						
Protein	7241	7241	7241	7241	7241	7241
Carbohydrate	322	322	322	322	322	322
Ligand	12	9	5	9	9	9
Ion	1	1	1	1	1	1
Solvent	1533	1633	1537	1497	1566	1457
RMS Deviations						
Bond length (Å)	0.006	0.005	0.005	0.006	0.006	0.006
Bond angle (°)	0.995	0.964	0.973	1.062	1.076	0.990
B factor	30.5	31.0	29.1	36.8	34.1	31.3
Ramachandran plot: core, allow, disallow	96.4%, 2.9%, 0.7%	96.4%, 2.9%, 0.7%	96.9%, 2.4%, 0.7%	96.3%, 2.9%, 0.8%	95.3%, 3.9%, 0.8%	96%, 3.2%, 0.8%

Data were collected at APS beamline 24-ID and ALS beamline 4.2.2.

$R_{merge} = \Sigma_{i,h} \mid I_{i,h} - \langle I_h \rangle \mid / \Sigma_{i,h} \mid I_{i,h} \mid$ where $\langle I_h \rangle$ is the mean of the i observations of the reflection h.

$R_{work} = \Sigma||F_o| - |F_c|| / \Sigma|F_o|$. R_{free} is the same statistic, but calculated from a subset of the data (5%) that has not been used in refinement.

Supplemental Figure 5: Statistics of the X-ray crystallographic data.
